# Thirteenth International Foamy Virus Conference—Meeting Report

**DOI:** 10.3390/v17081071

**Published:** 2025-07-31

**Authors:** Arifa S. Khan, Martin Löchelt, Florence Buseyne, Ottmar Herchenröder, Dirk Lindemann, William M. Switzer, André F. A. Santos, Marcelo A. Soares

**Affiliations:** 1 Laboratory of Retroviruses, Division of Viral Products, Office of Vaccines Research and Review, Center for Biologics Evaluation and Review, U.S. Food and Drug Administration, Silver Spring, MD 20993, USA; 2 Research Topic Immunology, Infection and Cancer, Division Virus-Associated Carcinogenesis, German Cancer Research Center (DKFZ), 69120 Heidelberg, Germany; m.loechelt@dkfz-heidelberg.de; 3 Institut Pasteur, Unité d’Epidémiologie et Physiopathologie des Virus Oncogènes, Université Paris Cité, 75015 Paris, France; florence.buseyne@pasteur.fr; 4 Institute of Experimental Gene Therapy and Cancer Research, Rostock University Medical Center, 18057 Rostock, Germany; ottmar.herchenroeder@med.uni-rostock.de; 5 Institute of Medical Microbiology and Virology, Faculty of Medicine, Technische Universität Dresden, 01307 Dresden, Germany; dirk.lindemann@tu-dresden.de; 6 Laboratory Branch, Division of HIV/AIDS Prevention, National Center for HIV, Viral Hepatitis, STD, and TB Prevention, Centers for Disease Control and Prevention, Atlanta, GA 30329, USA; bis3@cdc.gov; 7 Department of Genetics, Universidade Federal do Rio de Janeiro, Rio de Janeiro 21941-902, Brazil; afsantos.ufrj@gmail.com; 8 Oncovirology Program, Instituto Nacional do Câncer, Rio de Janeiro 21941-902, Brazil

**Keywords:** foamy virus, spumaretrovirus, New World primates, cross-species transmission, zoonosis, restriction factors, immune responses, FV vectors, virus replication, structural biology, virus–host co-evolution, latent infection

## Abstract

The 13th International Foamy Virus (FV) Conference was held from 8 to 10 November 2023 at the BioParque/Zoological Garden in Rio de Janeiro, Brazil. This was the first conference on spumaretroviruses to be held in the Southern Hemisphere and in the unique environment of the rainforest. New developments and current perspectives in FV research were presented. Highlights of the conference included the structural biology of the envelope protein (Env) and insights into its function and evolution, epidemiologic identification of Amazonian indigenous people with a high prevalence of simian FV (SFV) infections, investigations of virus biology and genomics using synthetic FV DNAs, studies of humoral immune response, and development and applications of SFV vectors. The last day of the meeting was a special tour of the Centro de Primatologia do Rio de Janeiro, located northeast of Rio de Janeiro amidst the protected rainforest, where New World primate hosts of spumaretroviruses are rescued and studied. Our report summarizes the meeting highlights and outcomes for future discussions.

## 1. Introduction

The International Foamy Virus (FV) Conference is an in-person meeting to provide an open platform for scientists from various institutions to present and discuss their latest research on FVs and create future collaborations to advance the field. Although much progress has been made in understanding the biology, molecular biology, genomics, and transmission of FVs, there is still a critical need to understand why FV is the only complex retrovirus without an associated disease [1], particularly in the case of simian foamy viruses (SFVs), which naturally occur in all non-human primate species and can infect humans by cross-species transmission from infected animals.

**Figure 1:**
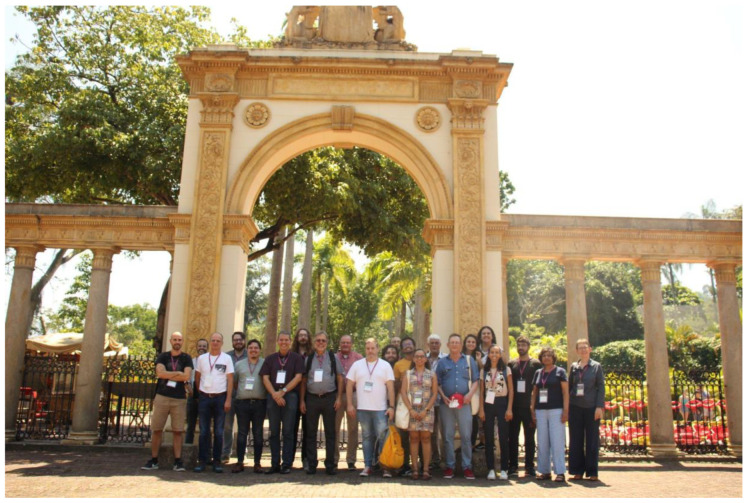
BioParque of Rio de Janeiro, the major venue for the 13th International Foamy Virus Meeting.

The 13th meeting was organized by Marcelo A. Soares and hosted by Mirela D’arc F. da Costa, Francine Bittencourt Schiffler, and André Felipe A. dos Santos at the BioParque do Rio de Janeiro/Zoological Garden, next to the Brazil Museu Nacional da Universidade Federal do Rio de Janeiro (Figure 1). During the conference, a visit to the BioParque veterinary clinic by Dr. Bruna Barbosa provided the unique opportunity for the conference participants to be informed of important aspects about mostly endangered host species for New World primate (NWP) FVs.

In his welcome address, Marcelo A. Soares pointed out that this was the first International Foamy Virus Conference that did not take place in the U.S. or Europe but was organized in the Southern Hemisphere.

## 2. Summary of Scientific Sessions

### 2.1. Epidemiology of Natural and Zoonotic Infections (Session Chair: Florence Buseyne, Institut Pasteur, France)

This session included four presentations that described infection of FVs in NWPs and one that described infection in humans exposed to SFV-infected NWPs in South America. SFVs infect a wide range of NWP species [2], and approximately one in five individuals occupationally exposed to infected NWPs develop antibodies against SFV [3]. The epidemiology of SFV in NWPs has generally been conducted on captive animals, and only five complete genome sequences from SFVs infecting NWPs are available.

To further understand NWP SFV transmission, four scientists from the Universidade Federal do Rio de Janeiro used semi-invasive swab sample collection to facilitate the testing of free-living and captive NWPs. The experimental procedure started with genomic DNA extraction and quality assessment by polymerase chain reaction (PCR) amplification of a cytochrome B gene fragment to confirm host species assignment. Then, SFV diagnosis was established using a quantitative PCR assay to amplify a 124-bp fragment of the retrovirus polymerase (*pol)* gene.

Matheus Augusto Calvano Cosentino presented the “Epidemiological Profile of Foamy Virus in Free-Living *Leontopithecus rosalia* (Golden lion tamarin) in the State of Rio de Janeiro, Brazil”. He showed data for this species that belongs to the *Callitrichidae* family and is endemic in the state of Rio de Janeiro. Swab samples from 79 animals were analyzed. The SFV prevalence rate was 43%, similar in both sexes, and increased with age. Of interest, this prevalence rate is the highest observed to date in free-living NWPs, which may be explained by interaction with other nonhuman primate (NHP) species, migration, and translocation of animals by humans.

Liliane T. F. Cavalcante presented on “Incidence of Foamy Virus Infection in an Invasive Neotropical Primate Species, *Leontopithecus chrysomelas* (Golden-headed lion tamarin) in the State of Rio de Janeiro, Brazil”. Data were presented on swab samples analyzed from 33 animals. The SFV prevalence rate was 18% and similar in both sexes. Captive animals from the same species have been previously studied with the same methodology [4]. The SFV prevalence rate was 25% in newly captured animals and 55% after six months of captivity. This study highlights how human intervention for wildlife management may impact SFV epidemiology in NWPs.

Amanda de Lucas Coimbra presented the “Evaluation of Use of Fecal Samples from captive and free-living muriquis (*Brachyteles* species (spp.)) for SFV diagnosis by real-time PCR”. These arboreal NWPs, belonging to the *Atelidae* family, are critically endangered, which precluded their capture for oral sample collection. Therefore, feces were used as the biological material for this study. None of the 56 samples analyzed tested positive for SFV, suggesting that low copy numbers or the presence of PCR assay inhibitors may have resulted in false negative results.

Gabriel Medeiros Viana presented the “Prevalence of Simian Foamy Virus in Captive *Alouatta* spp. (howler monkeys) from Santa Catarina, Brazil”. The talk focused on the Southern brown howler (*Alouatta guariba clamitans*), a subspecies from the *Atelidae* family. Oral swab samples were collected from 35 captive animals. The SFV prevalence rate was 21%, which was lower than in a previous study of free-ranging and captive *Alouatta* monkeys from different subspecies [2].

William M. Switzer (Centers for Diseases and Prevention, Atlanta, GA, USA) presented on “Endemic Foamy Virus Infection among South American Indians”. He presented the screening of >2500 archived sera collected in 1966–1997 from approximately 2000 individuals belonging to 18 indigenous tribes living in Brazil or in Venezuela. Both SFV and HTLV-2 infections were tested by validated serological methods. Mean prevalence of HTLV-2 was 6.1%, with familial clustering observed in two tribes, supporting secondary transmission. Mean prevalence of SFV was 9.5%, with familial clustering observed in seven tribes. Thus, Amerindians may be the first endemic SFV population, with data supporting NWP-to-human and possible human-to-human transmission [5]. The strength of the work was the number of tested samples and the rigorous serological screening with appropriate controls, using two enzyme immunoassays and one western blot assay. One caveat was the lack of stored samples of cells to confirm SFV diagnosis by PCR. If confirmed in an independent cohort with both serological and molecular diagnosis assays, these data will change the current paradigm that states the lack of human-to-human SFV transmission.

Together, the presentations on SFV epidemiology in NWPs and humans highlighted the high level of SFV circulating in South America and the public health importance of gaining further knowledge on the biology and transmission of the viruses.

### 2.2. Interactions of Foamy Viruses with the Immune System (Session Chairs: William M. Switzer, Centers for Disease Control and Prevention, Atlanta, GA, USA)

This session included two presentations that focused on determining components of the immune response responsible for suppression of viral replication within SFV-infected humans that could be responsible for the observed lack of disease and person-to-person transmission following zoonotic infections. Florence Buseyne (Institut Pasteur) presented “Neutralization of Zoonotic Simian Foamy Virus: Epitopes are Located in the Receptor Binding Domain”. She examined the location of neutralizing activity in the variable region of the surface protein (SUvar) of the envelope (Env) gene using plasma from 20 persons from Cameroon infected with gorilla SFV. These persons were previously shown to possess specific neutralization antibodies (nAbs) that blocked cell-free viral infectivity *in vitro* in the SUvar region of Env [6]. The study utilized a competitive neutralization assay (CNA) with soluble recombinant SU proteins that competed with SU on FV vector particles to identify the nAb targets. Mutations in the functional regions of the receptor binding domain (RDB), glycosylation sites, and genotype-specific sequences in the SU identified three conformational epitopes targeted by the nAbs using the CNA. The nAbs bound to peptide loops in the upper RBD subdomain, near the heparin binding site, and a region near the conserved N8 glycosylation site. These findings suggested that conformational changes in the RBD responsible for trimer formation that are crucial for receptor recognition, membrane fusion, and virus entry are targeted by SFV nAbs and limit transmission and control viremia within the infected host. Further, the results indicated that glycan shielding did not occur to facilitate immune escape. Additional details of the study have been recently published [7].

Thamiris Miranda (Universidade Federal do Rio de Janeiro) presented on “Assessment of Seroactivity to Ape SFV Env B-cell Epitopes among Brazilian Primates and Primate Handlers”. The study used an ELISA developed with peptides spanning the N96-V110 amino acids of the Env gene for SFV from an *Ateles* species (spider monkey), *Callithrix jacchus* (common marmoset), *Sapajus xanthosternos* (Golden-bellied capuchin), and *Saimiri sciureus* (common squirrel monkey) to check for recognition of the putative NWP SFV immunodominant epitope located in the surface (SU) Env peptide in serum samples from infected NWPs and humans. This same Env region is immunodominant in humans infected with SFV from Old World primates (OWP) [6,7,8]. The study used sera from 32 NWPs and from 56 workers exposed to NWPs in a Brazilian research center and a zoo with known SFV antibody status. No seroreactivity was found to the Env peptides with the NWP sera, while a moderate sensitivity (59–63%) and relatively high specificity (86–89%) were observed with the human sera. These observations suggested that a different immunodominant region of Env may exist for NWP SFVs and that the N96-V110 peptide is recognized less by persons with NWP SFV infection than those with OWP SFV infection. Additional studies are required to identify the NWP immunodominant peptides in NWP SFV infection.

### 2.3. Development and Applications of SFV Vectors and Molecular Clones (Session Chair: Marcelo A. Soares, Instituto Nacional do Câncer, Rio de Janeiro, Brazil)

This session included a presentation by Dirk Lindemann (Technische Universität Dresden, Dresden, Germany) entitled “TraFo CRISPR: Efficient Delivery and Transient Provision of Genome Engineering Tools by FV vectors”. He described a new delivery system based on prototype foamy virus (PFV) that allows full transient delivery of the CRISPR Cas9 genome engineering tools to target tissues. The major improvement compared to the first generation of the vector system is the employment of a new combinuclease with both RNA- and DNA-processing capabilities [9]. Use of the combinuclease enabled pre-single-guide RNA (pre-sgRNA) transcription from RNA polymerase II expression cassettes and its subsequent processing into mature Cas9 sgRNAs. Furthermore, it resulted in an efficient assembly of functionally active Cas9/sgRNA ribonucleoprotein (RNP) complexes within the vector packaging cells. Finally, data were presented suggesting that gene editing in target cells is largely mediated by vector-mediated delivery of functional Cas9/sgRNA RNPs rather than delivery of the individual components and de novo assembly of functional RNPs within the target cell.

Arifa S. Khan (U.S. Food and Drug Administration, Silver Spring, MD, USA) presented the “Molecular and biological characterization of a synthesized, infectious DNA of simian foamy virus serotype 1 (SFVmcy_FV21)”. Her presentation described the genomic characterization and infectivity analysis of a synthetic cloned plasmid DNA of SFV-1 and comparison to the parent virus stock. Sequencing and variant analysis of the pSFVmcy_FV21 virus indicated the absence of genetic diversity after recovery from *Mus dunni* cells, in contrast to the parent stock, which was a mixed population, with genome variants distributed across the genome. Infectivity studies comparing pSFVmcy_FV21 and the parental virus stock were conducted in different cell lines. Overall, the results were similar except in the Vero cell line, where both virus stocks showed slower replication kinetics and lower infectious virus titers than in the other cell lines. Nevertheless, pSFVmcy_FV21 showed a faster progression of cytopathic effects compared to the parent virus. Variant analysis after replication in Vero cells showed a significant increase in the presence of G-to-A mutations in the transcription transactivator (Tas) and between transactivator and envelope (Bet) genes for both virus stocks. Most of the mutations were stop codons resulting in a defective Tas protein and consequently a replication-incompetent virus, which could explain the low infectious virus titers obtained for both stocks in this cell line. These G-to-A hypermutations were likely the result of APOBEC3 activity on the replicating viruses. The high number of variants in the SFVmcy_FV21 mixed virus population may cause the slower CPE progression for SFVmcy_FV21 in Vero cells.

### 2.4. Structural Studies of Foamy Virus Proteins (Session Chair: Ottmar Herchenröder, Rostock University Medical Center, Rostock, Germany)

This session included a talk by Thomas Calcraft (Francis Crick Institute, London, UK) entitled “Integrated Structure of Prototype Foamy Virus” that described the integrated 3D structure of complete PFV virions, the first within the family of retroviruses, obtained by a combination of cryo-electron microscopy and cryo-electron tomography techniques. His presentation included details of the capsid’s native icosahedral structure, which is assembled as a fullerene shell via extensive Gag capsid domain interactions resulting in a much smoother and flatter capsid than that of orthoretroviruses. Furthermore, the first high-resolution 3D structure of glycoprotein complexes (GPCs) and GPC lattices embedded in the lipid bilayer of PFV virions were presented. The GPC structure unexpectedly revealed similarity to paramyxo- and pneumovirus F glycoproteins, suggesting that an ancient class 1 membrane fusion module was acquired by diverse groups of enveloped viruses. Surprisingly, the high-resolution structure of the GPC lattices observed on PFV virion surfaces revealed a strand-exchange mechanism where N-terminal residues of the envelope SU subunit extend into the neighboring GPC trimer within the lattice. Further details of this study have been recently published [10].

Ignacio Fernández (Institut Pasteur, Paris, France) presented “Structural Insights into the Foamy Virus Cell Entry”. His presentation described their recently published [11] 3D structure of the monomeric Env RBD of the gorilla FV. It is distinct from RBD structures in orthoretroviruses and comprises of two subdomains and an unexpected fold within the RBD. A model was proposed for the organization of the RBDs in the trimeric Env, indicating the upper region forming a cage-like structure at the apex of Env. Furthermore, it identified residues in the lower region as critical for the interactions of the RBD and viral particles with heparan sulfate that are important for infectivity, a function that could be experimentally verified. In addition, the 3D structures of the gorilla FV ectodomain in its pre- and post-fusion states were presented, which largely confirmed their model of pre-fusion state RBD organization in FV glycoprotein complex (GPC) [11]. The 3D ectodomain structures also revealed unexpected structural similarities to the fusion proteins of paramyxo-, pneumo-, and coronaviruses, providing a possible evolutionary link between these viral families and FVs. Finally, a mechanistic model was proposed for the FV GPC conformational change from the pre- to the post-fusion state that highlighted how the interplay of its structural elements could drive membrane fusion [12].

### 2.5. Molecular and Cellular Biology of Foamy Viruses (Session Chair: Arifa S. Khan, US Food and Drug Administration, Silver Spring, MD, USA)

This session included a presentation by Martin Löchelt (German Cancer Research Center (DKFZ), Heidelberg, Germany) entitled “High Titer Bovine Foamy Virus Variants: Selected Adaptive Changes in the Viral Integrase and LTRs”. Bovine foamy virus (BFV) replication is highly cell-associated; thus, it is mainly propagated by co-culture techniques. The presentation described new data obtained during an *in vitro* evolution and selection screening in bovine and hamster cell lines to obtain BFV variants with enhanced cell-free transmission. BFV variants were obtained, which were biologically and genetically characterized and molecular clones were generated. In addition to consistent changes in Gag and Env, which contribute to FV budding, base exchanges and specific duplications and deletions were noted in the viral long terminal repeat (LTR) regions [13,14]. More surprisingly, in-frame deletions of a cysteine-rich segment in the C-terminus of integrase occurred independently in bovine and hamster cells, removing overlapping sequences of different sizes in these viable BFV high-titer-adapted variants. Studies have been initiated to characterize and understand the phenotype of these adaptive changes.

Paul Lesbats (Université de Bordeaux, Bordeaux, France) presented “Timely Chromatin Invasion During Mitosis Governs Prototype Foamy Virus Integration Site Selection and Infectivity”. He described that PFV Gag protein interaction with a nucleosome H2A-H2B acidic patch is a major determinant for integration site selection and integration efficiency. Amino acid substitutions of highly conserved residues of a Gag chromatin binding site (CBS) showed a decreased interaction with host chromatin and led to an untimely chromatin capture during cellular mitosis. This tethering delay is accompanied by a decrease in integration efficiency as well as a redistribution of integration sites, notably toward markers associated with the late replication timing of host chromosomes. The study brings mechanistic evidence for this delayed chromatin binding caused by the Gag mutations not permitting H4-mediated nucleosome–nucleosome interactions to break within the acidic patch [15].

### 2.6. ICTV and the Binomial Nomenclature for Foamy Viruses (Session Chairs: Dirk Lindemann, Florence Buseyne, Martin Löchelt, and Arifa S. Khan)

This session discussed the new International Committee on Taxonomy of Viruses (ICTV) proposal for FVs. There was consensus for the need for binomial species names for FVs for alignment with the recent updates established by ICTV for other retroviruses. The FV taxonomy was updated in 2018 with new genera and species and a new virus nomenclature [16]. The species’ names have recently been updated to a binomial format, as required for the family *Retroviridae* by the ICTV [17]. It should be noted that the foamy virus names and abbreviations remain the same, except for the abbreviation for simian foamy virus *Ateles* spp. where the precise species is unknown (previous designation, SFVa*xx;* GenBank accession number EU010385; new designation, SFVa).

### 2.7. Historical and Future Aspects of Foamy Virus Research (Session Chair: Dirk Lindemann)

Ottmar Herchenröder (Rostock University Medical Center, Rostock, Germany) presented “A Narrative Story About Early Research on a Very Special Retrovirus”. He described the early days of FV research and the very basic methodologies of molecular biology used during that time, without any “omics” or other advanced techniques. He narrated how scientists convincingly proved that there is no bona fide human FV, while SFV may infect humans. The important molecular differences between FVs and the majority of conventional retroviruses were highlighted. For example, in contrast to other retroviruses, FV particles contain almost completely reverse transcribed double-stranded DNA. After infection and proviral integration into the host genomic DNA, transcription is initiated from an internal promoter, leading to early Tas protein expression. Finally, he commemorated scientists who had mentored some of the conference participants, thereby promoting successful careers. Taken together, the presentation highlighted “where we were” to realize “how far we have come” in the international FV research community, now being endowed with advanced technologies to gain further insights into these most ancient of all retroviruses.

Martin Löchelt (German Cancer Research Center, Heidelberg, Germany) presented “The Past, Present and Future of Foamy Virus Research—A Personal Perspective”. He described the path of FV research with respect to state-of-the-art topics and state-of-the-art experimental methodologies and how these aspects extrapolate into future directions. Personal concerns were also presented related to intrinsic and extrinsic factors that may influence the future of FV research, such as limited funding (particularly for studying non-pathogenic viruses), increasing focus on translational research versus basic sciences, and potential difficulties in resources for updating laboratories with advanced and complex high-throughput technologies, all in a context of concerns for a reducing community in the field.

## 3. Keynote Lectures

The first keynote speaker was introduced by Marcelo A. Soares (Instituto Nacional do Câncer, Rio de Janeiro, Brazil). Matias Melendez (Instituto Nacional do Câncer, Rio de Janeiro, Brazil) presented “Hybrid Viral Vectors for Biotechnology Applications”. He described how hybrid viral vectors have emerged as relevant tools in biotechnology, offering a versatile platform for a wide range of applications, from gene therapy to vaccine development. The herpes simplex virus (HSV) was illustrated as a great candidate for hybrid vector design. It was noted that HSV offers distinct advantages for biotechnological purposes, including a high transduction efficiency (including in central nervous system cells), a large cargo capacity (among the largest human DNA viruses, HSV can carry up to a 150 kb of foreign DNA), and the fact that the virus does not integrate into host chromosomes, thus reducing the risk of insertional mutagenesis. The presentation highlighted that hybrid vectors can enable enhanced transgene delivery and regulation, making them valuable tools in gene therapy and vaccine development.

André Felipe dos Santos (Universidade Federal do Rio de Janeiro, Rio de Janeiro, Brazil), introduced the second keynote speaker. Renato S. de Aguiar (Universidade Federal de Minas Gerais, Belo Horizonte, Brazil) who presented “The role of Endogenous Retroviruses and Other Retroelements in Health and Disease”. Human endogenous retroviruses (HERVs) are retroviral sequences integrated into the human genome and remnants of ancient retroviral infections. HERVs have been reported to play roles in neurogenesis, immune activation, and neurological disorders. Arboviruses often cause encephalitis, but their interactions with HERVs remain unclear. RNAseq data was presented from human primary astrocytes infected with the arboviruses including Zika (ZikV), Mayaro (MayV), Oropouche (OroV), and Chikungunya (ChikV) and analyzed using the TELESCOPE v. 1.0 software package. It was shown that MayV and ChikV displayed common HERV modulation, suggesting conserved transcriptional mechanisms. Fifteen HERVs, including the highly upregulated HERV4_4q22, were co-modulated by all four arboviruses. Nearby gene upregulation was observed, with 14 genes common to all four infections and 93 between MayV and ChikV that were linked to processes like cell replication, cytoskeleton dynamics, vesicle trafficking, and antiviral response. These findings suggest HERV involvement in gene regulation during arboviral infections. More details of the study have been published [18].

## 4. Visit to the Centro de Primatologia do Rio de Janeiro

**Figure 2:**
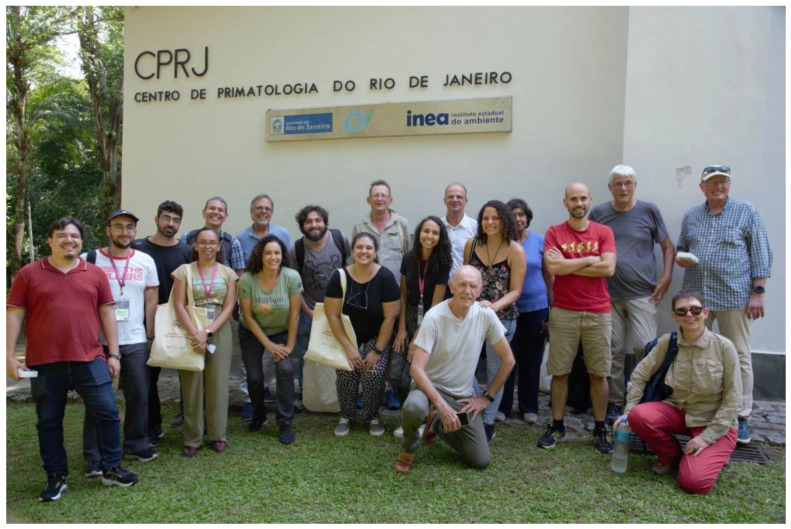
Centro de Primatologia of Rio de Janeiro at Guapimirim city, an organization by Instituto Estadual do Ambiente (State Institute of Environment), where the last day of the event occurred. Dr. Alcides Pissinatti, the Director of the Center, is in the center of the picture.

The conference ended on Friday, Nov. 10th with a visit to the Centro de Primatologia do Rio de Janeiro, with Dr. Alcides Pissinatti as the facility director (Figure 2). This was located close to Guapimirim, in the protected area called *Paraíso* Ecological Station, in the Atlantic Forest ecosystem. A guided tour through the station revealed the impressive diversity of NWPs, some of which were donors of SFV isolates.

## 5. Conclusions

The 13th International FV meeting provided an exciting and unique setting with highly productive scientific exchange and discussions. The guided visit to BioParque and the Rio de Janeiro Primate Center including the Center Museum and Primate Vivaria was a unique opportunity for the participants to visit the NWPs that are the natural hosts for SFVs. The FV community and other researchers are encouraged to participate in the 14th International FV Conference, to be held in Bordeaux, France on 22–25 September 2025, to hear more on the innovations and advances in spumaretrovirus research. More information can be obtained from the conference host: paul.lesbats@u-bordeaux.fr.
